# Factors of choking under pressure in musicians

**DOI:** 10.1371/journal.pone.0244082

**Published:** 2021-01-06

**Authors:** Shinichi Furuya, Reiko Ishimaru, Noriko Nagata

**Affiliations:** 1 Sony Computer Science Laboratories Inc. (Sony CSL), Tokyo, Japan; 2 Sophia University, Tokyo, Japan; 3 School of Science and Technology, Kwansei Gakuin University, Sanda, Japan; Medical University of Vienna, AUSTRIA

## Abstract

Under pressure, motor actions, such as those required in public speech, surgery, or musical performance, can be compromised, even when these have been well-trained. The latter is often referred to as 'choking' under pressure. Although multifaceted problems mediate such performance failure in anxiogenic situations, such as compromised motor dexterity and cognitive disruption, the fundamental set of abnormalities characterizing choking under pressure and how these abnormalities are related have not been elucidated. Here, we attempted, first, to classify behavioural, psychological, and physiological abnormalities associated with choking under pressure in musicians and, second, to identify their relationship based on datasets derived from a questionnaire with 258 pianist respondents. Explorative factor analysis demonstrated eight functional abnormalities related to the musicians' choking, such as attention to the audience, erroneous motor actions, perceptual confusion, and failure of memory recall, which however did not include exaggerated attention to the performance. This suggests distraction of attention away from skill execution, which may underlie the spoiled performance under pressure. A structural equation analysis further inferred causal relationships among them. For instance, while failure of memory recall was influenced by passive behaviours manifesting under pressure, erroneous motor actions during performance were influenced by feeling rushed and a loss of body control. In addition, some specific personal traits, such as neuroticism, public self-consciousness, and a lack of confidence, were associated with the extent to which pressure brought about these abnormalities. These findings suggest that distinct psycho-behavioural abnormalities and personal traits underlie the detrimental effects of pressure on musical performance.

## Introduction

The performance of highly trained skilled behaviours in public sometimes goes worse than it does during practice due to psychological stress, and this phenomenon is called choking under pressure [1]. Choking under pressure triggered by such stress accompanies heterogeneous abnormalities in motor, sensory, and cognitive skills and brings about abnormal ways of thinking and malfunctions in the autonomic nervous system. These malfunctions include but are not limited to failure of memory recall [2], loss of motor dexterity [3–6], atypical attentional control [7], aberrant elevation in heart rate [8–10], and muscular stiffness [5]. However, the fundamental set of psychological, physiological, and behavioural abnormalities characterizing choking under pressure and their relationship have not been elucidated. It also remains unknown in what manner detrimental effects of choking under pressure on the skilful behaviours are related to the innate personal traits of the individual. Resolving these issues would be useful for performers such as, for instance, musicians, athletes, surgeons and politicians. In addition, examining the underpinning of choking would shed more light on the neuropsychological mechanisms behind poor or suboptimal performance.

Several theories have been proposed to account for choking under pressure [11–13]. Distraction theory and explicit monitoring theory propose abnormal attention during movements [2, 13, 14]. The distraction theory proposes that psychological pressure causes attentional shifts of performers away from skill execution toward task-irrelevant cues [7, 15]. Because this abnormal attentional shift has detrimental effects on working memory [16], choking under pressure often causes failure of memory recall [2, 6]. Neuroimaging studies also demonstrated abnormal functional connectivity between the dorsolateral prefrontal cortex [15] and inferior parietal cortex [17, 18], which are regions responsible for cognitive functions such as working memory and attention, and the motor cortex, which plays a role in the precision of skilled movements. Furthermore, the successful recall of procedural memory relies on sensory feedback that matches produced motor actions [19–21], which implicates the significance of perception in preventing failure of memory recall. In addition, the distraction-related choking can be associated with excessive arousal and motivation induced by incentives such as monetary reward and social evaluation [22, 23]. Reward neural circuits mediate contrasting effects of low and high incentives on performance [22, 23], which explains an inverted-U-shaped relationship between the arousal level and performance [24]. An increased sensitivity to incentives under pressure can also affect decision making processes, such as whether performers take or avoid risks. The explicit monitoring theory posits that after an appraisal of performance pressure, attention shifts exaggeratedly toward the procedural process of motor performance, which eventually collapses skilful performance [1, 2, 14]. Particularly in trained individuals such as sports athletes and musicians, unnecessarily excessive attention drawn to skill execution disrupts the procedural process that normally runs automatically, which results in loss of fine motor control [14, 25]. The loss of fine motor control involves the spatiotemporal imprecision of skilled actions, such as speeding up one’s movement tempo unintentionally. A recent study demonstrated that an abnormally increased tempo during a piano performance under pressure was associated not with an abnormally elevated heart rate but instead with a lack of coordinated movement between the fingers [6]. The abnormally increased co-activation of an antagonistic pair of muscles due to psychological stress can reflect a strategy to compensate for this motor imprecision [5, 26], which also has potential risks of affecting recall of proceduarized memories via the feedback control [27]. Finally, a schema theory can be also associated with choking under pressure, in a way that recalling motor memories that were encoded in a specific situation (e.g. practice room) can be disrupted in an unfamiliar situation such as a concert hall [28]. Importantly, this theory and the others are not necessarily exclusive, because for instance, the disruption of memory recall can elicit abnormal attentional shift. Together, although previous studies demonstrated the complexity of factors of choking under pressure, we postulated that candidate factors are abnormalities of attentional control, fine motor control, perception, memory recall, and decision making.

The present study aimed to identify a fundamental set of factors characterizing choking under pressure in musicians and to understand their relationship. Musicians are individuals who commonly suffer from choking under pressure [29–33]. A recent meta-analysis based on case reports, intervention, cross-sectional, and cohort studies reported that the prevalence of music performance anxiety ranged from 17% to 60% [30]. Nevertheless, factors of choking under pressure in musicians and a relationship between the factors have been not yet fully elucidated [34]. Specifically, we attempted to understand whether the distraction theory or explicit monitoring theory explains the musicians’ choking. Also, there are individual differences in the problems linked to choking under pressure that could be related to personal traits. For example, musicians’ anxiety is known to be associated with neuroticism [32], which can be linked to the culture of music (e.g. teaching and learning). Some of the candidate factors of choking under pressure are related to personal traits such as between attentional control and neuroticism [35, 36], which let us postulate a relation between personal traits and choking under pressure in musicians. To shed light on traits particularly related to individual psychological, behavioural, and physiological abnormalities under pressure is needed to prevent music performance anxiety in a client-tailored manner and to optimize interventions for it, such as cognitive behavioural therapy, coaching, and specialized training designed for musicians. In addition to defining factors characterizing choking under pressure in musicians, the present study, thus, also attempts to address personal traits associated with choking under pressure in musicians.

## Methods

### Participants

We administered an online questionnaire to 300 pianists who were receiving or had received a professional education in playing the piano at a musical conservatory and/or privately (= an inclusion criteria) without any histories of being diagnosed as psychiatric disorders (= an exclusion criteria), and 258 of them completely responded to all questions (218 women and 40 men, all aged over 18 years old) in a complete manner (i.e. respondents who did not respond to all questions were excluded from the subsequent analyses) within a period of 6 months (from the beginning of July to the end of December in 2017). This sample can be considered representative of a larger population because of no biased distribution of the questionnaire. The pianists were identified based on the history of their past piano education and performance, which were described in the online questionnaire. The return rate was 86%. All respondents were adult Japanese pianists with an average age of 27.6 ± 11.2 years old (mean ± SD). The mean duration of piano playing was 20.8 ± 9.6 years. Among respondents, 231 were receiving or had received professional education in piano playing at musical conservatories, 179 have received prizes at some domestic piano competitions, and 32 have won prizes at international piano competitions. Although none of the respondents used medications for psychiatric disorders, we included musicians diagnosed with previous anxiety-stress-depression states (n = 6). On the questionnaire, pianists received information guaranteeing that all data were collected anonymously and confidentially (i.e., no personal data was included that would enable the identification of the pianists). Prior to starting the questionnaire, an informed consent was obtained from each respondent. In accordance with the Declaration of Helsinki, the study procedures were explained to all participants prior to responding to the questionnaire. The whole experiment protocol was approved by the ethics committee of Sophia University (2017–69).

### Questionnaire

The present questionnaire consisted of a total of 93 questions that were classified into 2 categories according to their contents (S1 File). The first category (64 items) asked about physical, mental, and emotional experiences specifically when playing challenging pieces that were well-practiced and prepared on stage in front of audience (i.e., concert, audition, and competition) (i.e., “choking under pressure experiences”). The question items were created based on a questionnaire designed for assessing choking under pressure in athletes [37, 38]. These questions included physical sensation; physiological responses; memory; attention; awareness; self-consciousness; confusion of sensory, motor, and cognitive skills; fatigue; behavioural strategies; and feelings of physical and mental imperfection. We adopted questionnaire items that were used to assess choking under pressure in athletes because our target was detrimental effects of psychological stress on sensory, motor, and cognitive functions subserving skilful musical performance. We also carefully selected the questionnaire items so that they can test the hypotheses raised based on previous studies. We pretested the questionnaire with another forty pianists and ensured that the questionnaire items covered their experience of choking under pressure by confirming reliability of the answers through a test-retest evaluation with an interval of one month. At this pre-test, all of the questionnaire items were provided, and the order was randomized between the test and retest. The respondents were also asked to rate the severity of their choking under pressure, which was defined as failure of the execution of a well-learned, proceduralized skill under high pressure when the desire for superior performance is maximal and yields poorer outcomes than would otherwise be expected [1]. The second category (29 items) asked about personal traits, particularly concerning neuroticism and extroversion, on the basis of previous reports that these traits are associated with choking under pressure in athletes [1, 37] (i.e., “personal traits”). Again, the question items were created based on a questionnaire designed for assessing choking under pressure in athletes [37, 38]. The questions included neuroticism, inter-personal positiveness, introversion, public self-consciousness, and lack of confidence. All questions were rated on a five-point Likert-type scale. The question items are summarized in the S1 File.

### Data analysis and statistics

Using the rating results of the choking under pressure experiences, an exploratory factor analysis (maximum likelihood method, promax rotation) was performed to classify physical, mental, and emotional experiences. Question items were eliminated from further analysis when the factor loading value was lower than 0.35 and when the factor was not retained again the rotation [39]. To develop a structural analytical model describing the relationship between the factors quantitatively, a covariance structure analysis was performed. In this analysis, paths between the factors were drawn based on the following criteria. First, observed and latent variables in the model were question items and factors identified by the factor analysis, respectively. Here, the question items used as the observed variables were all of those not excluded by the factor analysis. Second, a path between the factors in which the correlation coefficient was significantly higher than 0.4 was used. Third, because both erroneous motor actions and failure of memory recall are problems that the present study particularly focused on, we assumed these factors had influences on but no outputs to the other factors. Finally, cause-and-effect relationships were postulated based on previous studies on choking under pressure [2, 6, 37, 40]. The fitting accuracy was evaluated according to the SRMR (standardized root mean square residual), CFI (comparative fit index), and RMSEA (root mean squared error of approximation). The statistical significance level was set to 0.05. Factor analysis and covariance structure analysis were performed using SPSS (ver. 24, IBM Inc.) and Amos (ver. 24.0, SPSS), respectively. Similarly, factor analysis was also performed to classify the items regarding personal traits.

## Results

### Characteristics of music performance anxiety

#### Exploratory factor analysis

Among the 64 items addressing choking under pressure experiences of musicians (i.e. music performance anxiety), factor analysis identified 37 items and 8 factors based on the exclusion criteria. Table 1 summarizes the question items classified into each of the 8 factors and their factor loading values. Based on the items within the individual factors, each of the 8 factors was labelled as follows: “attention to the audience” (F1), “abnormal physical sensations” (F2), “erroneous motor actions during performance” (F3), “vicious cycle of choking under pressure” (F4), “perceptual confusion” (F5), “feeling rushed and out of body control” (F6), “failure of memory recall” (F7), and “passive behaviours” (F8). The cumulative contribution ratio for the 8 factors was 57.0%. Items that were excluded due to a low factor loading score involved paying too much attention to the performance itself, which included focusing too much on self-motion/posture during playing, feeling concern about a passage that is difficult to play, and paying too much attention to where to look while playing.

**Table 1:** Results of an exploratory factorial analysis of choking under pressure in musicians.

factor	item	F1	F2	F3	F4	F5	F6	F7	F8
F1 attention to the audience	attention to being evaluated by the others	**0.88**	-0.01	0.07	-0.12	0.04	-0.04	-0.06	0.00
	awareness of being in front of acquaintances	**0.79**	-0.05	0.00	0.08	0.02	-0.17	0.10	0.01
	awareness to be in front of a lot of audiences	**0.77**	-0.04	-0.05	0.00	0.04	0.00	-0.03	0.06
	feeling of psychological pressure from audiences	**0.55**	0.03	0.09	0.01	-0.08	**0.34**	-0.06	-0.11
	sensation that all audiences focus only on oneself	**0.51**	0.05	-0.14	0.07	0.16	0.12	0.00	0.05
F2 abnormal physical sensations	sensation of abnormal breathing	-0.06	**0.79**	0.04	0.09	0.06	-0.16	-0.06	0.03
	sensation of choking	-0.08	**0.74**	0.06	0.09	0.04	-0.16	-0.11	0.05
	sensation of muscular weakness	-0.04	**0.64**	-0.06	-0.05	**0.35**	0.07	-0.04	-0.04
	physical sensation of shaking	0.11	**0.45**	-0.01	-0.11	0.03	0.23	0.10	0.03
	being thirsty	0.08	**0.41**	0.05	-0.13	-0.07	0.16	-0.02	-0.01
	loss of tactile sensation of the hand	0.04	**0.40**	-0.03	-0.08	0.37	0.18	0.09	-0.13
F3 erroneous motor actions during performance	insufficient quality of performance	0.00	0.02	**0.79**	0.17	-0.06	-0.05	-0.12	0.06
	a gap between the ideal and real performance	0.06	0.06	**0.77**	0.04	-0.03	-0.03	0.06	0.07
	a loss of precision in performance	-0.07	0.02	**0.71**	-0.05	0.10	0.05	0.11	0.01
	anxiety of making pitch errors	0.14	0.03	**0.36**	0.09	-0.16	0.32	0.07	0.07
F4 vicious cycles of choking under pressure	augmentation of the anxiety due to a failure to cope with it	-0.01	0.04	0.14	**0.78**	0.02	-0.04	-0.04	0.04
	failure to calm down	0.01	0.12	0.08	**0.73**	0.08	0.07	-0.10	-0.11
	promotion of the anxiety due to failures to make desired performance	0.02	-0.01	0.06	**0.63**	0.20	0.09	-0.09	0.05
	promotion of the anxiety following making erroneous actions	0.02	-0.20	-0.08	**0.63**	0.09	0.15	-0.03	0.29
F5 perceptual confusion	difficulty in feeling the weight of the piano keys	0.01	0.07	-0.01	0.04	**0.68**	-0.12	-0.04	0.08
	abnormal visual sensations	0.10	0.07	-0.04	0.09	**0.55**	0.06	0.06	-0.15
	unstable feeling in the hands	-0.11	0.15	0.02	-0.18	**0.45**	**0.32**	0.05	0.12
	sensation that the environment hinders the performance	0.14	0.14	0.05	0.17	**0.44**	-0.23	0.11	-0.06
	abnormal weight percetion of piano keys	0.06	-0.04	-0.06	0.25	**0.40**	0.07	-0.04	0.09
	failure to predict upcoming sensations	-0.03	-0.15	0.20	0.23	**0.39**	0.17	0.10	-0.25
F6 feeling rushed and out of body control	feeling rushed	-0.10	0.07	-0.08	**0.44**	-0.18	**0.70**	0.11	-0.12
	feeling nervousness	0.06	0.23	-0.11	0.22	-0.29	**0.67**	0.01	0.00
	sensations of exaggerated muscular tension	0.03	-0.11	0.06	-0.11	0.16	**0.64**	-0.14	0.22
	an incapability of relaxing the muscles	-0.04	-0.10	0.07	-0.09	0.29	**0.59**	-0.15	0.19
	a loss of intended control of body movements	-0.12	0.07	0.27	0.06	0.15	**0.49**	-0.05	0.01
	a lack of temporal precision of movements	-0.02	-0.23	0.07	0.21	0.13	**0.46**	0.10	-0.11
F7 memory slip	forgetting a memorized performance	0.02	-0.15	0.07	-0.19	0.02	-0.02	**1.02**	0.05
	failure to recall a memorized performance	-0.04	0.06	-0.05	0.08	0.05	-0.04	**0.80**	0.03
F8 passive behaviours	avoidance of risk-taking during performance	0.04	-0.04	0.06	0.05	-0.02	0.05	-0.03	**0.73**
	inexpressive performance	-0.02	0.16	0.20	-0.10	-0.11	0.09	0.11	**0.47**
	delayed reaction to unexpected actions when playing	-0.04	-0.01	-0.10	0.29	0.13	-0.01	0.24	**0.39**
	failure to make quick decisions when playing	0.08	0.28	-0.04	0.19	-0.05	0.00	0.11	**0.37**

The number indicates a factor score.

The bolded number indicates a factor score larger than 0.30.

F1 (attention to the audience) consisted of 5 items, which were attention to being evaluated by others, awareness of being in front of acquaintances, and the sensation that all audiences focus only on oneself. F2 (abnormal physical sensations) had 6 items, which included sensations of abnormal breathing, shaking, choking, muscular weakness, and being thirsty. F3 (erroneous motor actions during performance) consisted of 4 items, which were insufficient quality of musical performance, a gap between the ideal and real performance, and a loss of precision in performance. F4 (vicious cycle of choking under pressure) had 4 items, which were the augmentation of the anxiety due to a failure to cope with it, failure to calm down, and the promotion of the anxiety following making erroneous actions. F5 (perceptual confusion) consisted of 6 items, which included difficulty in feeling the weight of the piano keys, abnormal visual sensations, unstable feeling in the hands, and failure to predict upcoming sensations. F6 (feeling rushed and out of body control) had 6 items, including feeling impatience and nervousness, sensations of exaggerated muscular tension and an incapability of relaxing the muscles, and a loss of intended control of body movements. F7 (failure of memory recall) included 2 items that consisted of forgetting and failure to recall a memorized performance. F8 (passive behaviours) consisted of 4 items, such as the avoidance of risk-taking during performance, inexpressive performance, delayed reaction to unexpected actions when playing, and failure to make quick decisions when playing.

#### Covariance structure analysis

**Fig 1:**
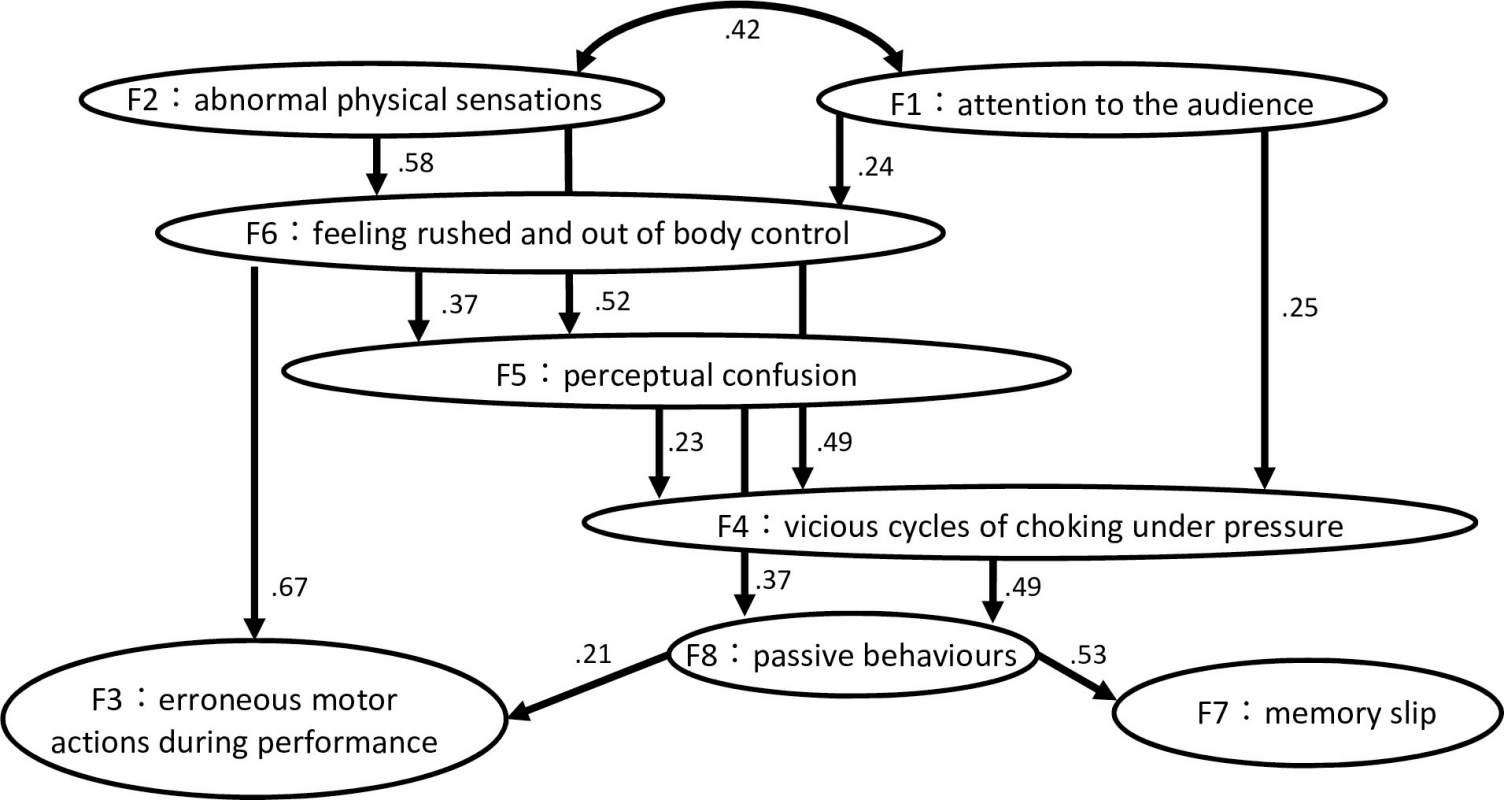
A schematic drawing of the results of a covariance structure analysis depicting inferred causal relationships between eight factors characterizing behavioural, physiological, and psychological abnormalities due to music performance anxiety. The number indicates a path coefficient between factors.

Fig 1 illustrates the results of a covariance structure analysis that was performed based on the results of the exploratory factor analysis. The SRMA, RMSEA, and CFI scores of the developed model were 0.07, 0.06, and 0.90, respectively. In this model inferring a causal relationship between the factors, F3 and F7 were factors without any output paths, whereas F1 and F2 were factors without any input paths. F3 had strong and weak input paths originating from F6 and F8, respectively. F7 had an input path only from F8. F8 had input paths from both F4 and F5. F4 had input paths from F1, F5, and F6, whereas F5 had input paths from F2 and F6. F6 had input paths from F1 and F2. Finally, there was a path bridging between F1 and F2.

### Personal traits

Factor analysis classified 28 of 29 question items regarding personal traits into 5 factors, which accounted for 53.0% of the total variance. Table 2 summarizes the results of the factor analysis. Each of these factors was labelled as follows: neuroticism (C1), inter-personal positiveness (C2), introversion (C3), public self-consciousness (C4), and lack of confidence (C5). C1 (neuroticism) consisted of 14 items, which included traits such as nervousness, oversensitivity, persistence on trivial issues, mental instability, likelihood/tendency to fatigue/experience guild, tension and anxiety about many issues. C2 (inter-personal positiveness) had 4 items: communication ability, activity level, friendliness, and openness towards strangers. C3 (introversion) included 3 items: gentleness, quiet, and taciturnity. C4 (public self-consciousness) consisted of 3 items: concerning about external evaluation, rumour, and public. C5 (lack of confidence) had 4 items: self-confidence, active speaking, positiveness, and passive traits.

**Table 2:** Results of an exploratory factor analysis of the personal traits.

factor	item	C1	C2	C3	C4	C5
C1 neuroticism	anxious about many issues	**0.78**	0.13	0.04	0.04	0.08
	nervousness	**0.75**	-0.06	0.03	-0.04	-0.24
	oversensitiveness	**0.74**	0.06	0.05	-0.04	0.13
	persistent on trivial issues	**0.72**	-0.03	0.02	0.09	-0.08
	negative thinking	**0.72**	0.14	0.11	-0.06	0.23
	serious thinking	**0.71**	0.06	0.14	-0.06	0.03
	mental fragility	**0.70**	0.06	0.01	0.18	-0.09
	anxious about unnecessary issues	**0.69**	0.14	0.00	-0.03	0.14
	mental instability	**0.62**	-0.04	0.04	0.08	-0.09
	easily gets tired	**0.61**	-0.30	-0.14	-0.10	-0.02
	tense	**0.52**	-0.03	-0.03	0.00	0.23
	easy to be distressed	**0.49**	-0.03	-0.23	-0.02	**0.39**
	easy to get irritated	**0.48**	-0.15	-0.08	-0.03	-0.05
	readiness of feeling guilty	**0.42**	0.00	0.00	0.20	0.18
C2 inter-personal positiveness	communicative	-0.07	**0.80**	0.10	0.03	-0.04
	friendliness	-0.01	**0.75**	-0.03	0.04	-0.02
	active	0.16	**0.52**	-0.15	0.00	-0.25
	openness towards strangers	-0.28	**0.40**	-0.12	-0.23	0.17
C3 introversion	gentle	0.02	0.12	**0.93**	-0.02	0.03
	quiet	0.03	-0.11	**0.72**	-0.02	-0.11
	taciturn	-0.08	0.27	**-0.59**	0.12	0.05
C4 public self-consciousness	concerning about external evaluation	0.07	0.05	-0.01	**0.80**	-0.05
	concerning about rumor	0.03	0.10	-0.03	**0.73**	0.07
	concerning about in public	0.00	-0.14	-0.07	**0.67**	0.09
C5 lack of confidence	self-unconfidence	0.16	-0.04	-0.08	0.05	**0.64**
	active speaking	0.27	0.28	-0.21	-0.17	**-0.41**
	positive	-0.29	0.27	0.05	0.11	**-0.39**
	passive traits	0.01	-0.19	0.29	0.06	**0.36**

The number indicates a factor score.

The bolded number indicates a factor score larger than 0.30.

To define a relationship between performance deterioration due to music performance anxiety (i.e., F3 and F7, which were factors without output paths in Fig 1) and personal traits, all respondents were divided into two groups according to whether the factor score for these two factors (F3 and F7) was higher or lower than 0 (note that the mean factor score was 0). Table 3 summarizes the mean factor score of each of the 5 trait factors for the two groups segregated according to the F3 and F7 factor scores. For each of the F3 (erroneous motor actions) and F7 (failure of memory recall) scores, two-sample t-tests with Bonferroni corrections for five comparisons (p = 0.01) demonstrated a significant group difference in the factor scores for C1, C4, and C5. This result indicates that respondents whose factor scores for F3 and F7 were higher exhibited higher tendencies of neuroticism, public self-consciousness, and lack of confidence.

**Table 3:** Results of two-sample t-tests of the personal-traits factor scores between two groups classified according to a score of each of the music-performance-anxiety factors.

		C1: neuroticism	C2: interpersonal positiveness	C3: introversion	C4: public self-consciousness	C5: lack of confidence
F3: erroneous motor actions	factor score (high group)	**0.2228**	0.0109	0.0105	**0.1715**	**0.1552**
	factor score (low group)	**-0.4920**	0.0672	-0.2373	**-0.4574**	**-0.6784**
	t value	-5.47*	0.59	-1.55	-4.79*	-7.41*
F7: memory slip	factor score (high group)	**0.1230**	0.0265	-0.0554	**0.1491**	**-0.0375**
	factor score (low group)	**-0.2893**	0.0431	-0.1342	**-0.3474**	**-0.3622**
	t value	-3.01*	0.21	-0.48	-3.88*	-2.73*

The factor score indicates one of the personal trait factors.

* indicates p < 0.01 (with Bonferroni corrections for 5 comparisons).

## Discussion

The present study demonstrated eight fundamental factors characterizing the choking under pressure in musicians. Overall, these factors can be classified into three categories: psychological (i.e., F1, F4, F6, F8), physiological (i.e., F2), and behavioural (i.e., F3, F5, F7) abnormalities. A relationship between these factors indicates that two problems that particularly lead to spoiled skilled performance (i.e., failure of memory recall and erroneous motor actions) were mediated by both shared and distinct factors. Failure of memory recall was influenced by passive behaviours manifesting under pressure, whereas erroneous motor actions during performance were influenced by feeling rushed and a loss of body control. Furthermore, personal traits such as neuroticism, public self-consciousness, and a lack of confidence were likely to be linked to these performance failures, which indicates that the extent to which the choking under pressure negatively impairs skilled musical performance depends, in part, on personal traits. While factors associated with choking under pressure in sports athletes have been investigated previously [11, 37, 38, 40, 41], to the best of our knowledge, this is the first study that identified eight specific factors related to choking under pressure in musicians, as well as their relationship.

Among various theories of choking under pressure, there are at least two theories accounting for the detrimental effects of pressure on performance according to abnormal attentional control. Psychological stress can either distract attention away from skill execution due to a shift in attentional focus towards task-irrelevant cues (i.e., distraction theory) [7, 15] or augment the explicit monitoring of skill execution, thereby disrupting its automated processes (i.e., explicit monitoring theory) [1, 2, 14]. Our results may support the former theory because attention to the audience (i.e. F1), but not too much attention to the performance itself (i.e. a factor discarded by the analysis), was identified as a factor characterizing music performance anxiety. Our observation is compatible with a finding that choking under pressure diminishes a functional connectivity between the motor cortex and prefrontal cortex responsible for the performance monitoring, which supports the distraction theory [15]. However, several studies reported that skill levels of performers are related to the pressure effects on attentional control [11, 25], which can confound the present results originating from pianists with different skill levels.

Our covariance structure analysis further exhibited a putative link between passive behaviours (F8) and both erroneous motor actions during performance (F3) and failure of memory recall (F7), which had no output path in this model. Passive behaviours included delayed reaction to unexpected actions when playing, inexpressive performance, and the avoidance of risk-taking during performance, which suggests abnormal decision making such as a risk avoidance behaviour. Interestingly, vicious cycles of choking under pressure (F4), which were characterized mainly by failure to cope with the pressure, were related to passive behaviours, which implies that the destabilizing effect of a loss of attentional and emotional control in performance underlies passive behaviour under pressure. In addition, perceptual confusion was also linked to passive behaviour, which is compatible with previous reports that the successful recall of procedural memory relies on sensory feedback that matches the produced motor actions [19–21].

Erroneous motor actions during performance under pressure (F3) were also linked to feeling rushed and out of body control (F6) in the present model. First, it is reasonable that feeling rushed resulted in out-of-tempo control, which is a typical behavioural abnormality experienced under pressure [6]. This can be also compatible with the polyvagal theory that proposes a relation between malfunctions of the autonomic nervous and choking under pressure [42]. Second, a loss of neuromuscular control, such as failure to relax the muscles, can elevate the viscoelasticity of the finger muscles, which increases biomechanical constraints on movement independence between the fingers due to anatomical connections across multiple fingers [43, 44]. Our recent study also demonstrated an association between reduced movement independence between the fingers and a loss of tempo control in piano performance under pressure [6]. Furthermore, movement independence between the fingers is responsible for the accuracy of force control in piano performance [45]. It is therefore plausible that choking under pressure degrades the control of independent movements between the fingers due to elevated muscular stiffness and, thereby, lowers the spatiotemporal precision of the performance. However, it should be emphasized that psychological pressure does not necessarily deteriorate the performance, as known by an inverted-U-shaped relationship between pressure and performance [24] or a phenomenon of flow [46]. In this respect, the present choking under pressure in musicians specifically argues detrimental effects of pressure on the performance.

Our factor analysis classified personal traits of the present pianists into five categories. Three among these five traits, which were neuroticism (C1), public self-consciousness (C4), and a lack of confidence (C5), were identified as factors accounting for the detrimental effects of music performance anxiety. Namely, pianists who were less anxious, nervous, and negative with higher self-confidence and less concern about external evaluation experienced failure of memory recall and erroneous motor actions in performance to a smaller extent. Both self-consciousness and a lack of confidence implicate traits concerning external evaluation, which indicates increased attention to task-irrelevant cues during the anxiogenic situation and, therefore, provides further supports for the distraction theory. A previous study also reported that neuroticism was a trait related to choking under pressure in musicians [32], which implies a relationship between some but not all trait anxieties and state anxiety of musicians.

There are some implications of the present observations for preventing the detrimental effects of choking under pressure on musical performance. First, psychological intervention, such as cognitive behavioural therapy [30] and writing about worries prior to a stressful evaluation situation [47], may aid in reducing the worries of the performers because some personal traits were related to music performance anxiety. However, such an intervention effect may depend on person and situation, which requires client tailored approaches to optimize interventions according to each performer’s goal. For it, in addition to the therapeutic approaches, coaching and mental training can be helpful as ways of providing strategies to cope with the pressure. Second, the acquisition of skills to reduce muscular tension in musical performance may lower the risk of performing erroneous motor actions when performing under pressure. For example, practising a sequence of movements with a variety of rhythms can reduce unnecessary muscular activities during piano playing [48], whereas excessive attention to temporal precision during piano practising prevents the reduction of such unnecessary muscular tension [49]. Third, a link of passive behaviours that include inexpressive performance to the performance failures concerns potential risks of taking medication that supresses the sympathetic nerve activities (e.g. beta blocker) in order to cope with choking under pressure in music performance, because it can further suppress expressive performance and thereby increase risks of making the performance failure.

Several limitations of the present study should be addressed because this is not an empirical study that investigated behavioural, physiological, and psychological responses to pressure. First, it is necessary to perform intervention studies to identify causal relationships between the abnormalities emerging under pressure, because the present statistical analysis merely inferred the causality under several prerequisites. Second, the present results are based not on a phenomenon that occurred under pressure but on subjective reports with respect to what pianists felt and remembered about their music performance anxiety. Third, the present conclusions are based on the results of skilled pianists, and so a future study should investigate music performance anxiety of unskilled players and other musicians. Fourth, the present study cannot rule out a sampling bias from a specific population (e.g. self-report ability, willingness to take part in the study) due to difficulty of controlling the participants (e.g. gender, level of expertise of participants). Fifth, the questionnaire items used in the present study were designed based on the questionnaire investigating choking under pressure in sports athletes, which limits the understanding of the whole aspects of choking under pressure in musicians (e.g. aesthetics expression under pressure). Sixth, although the questionnaire items were evaluated a priori through the pre-test with a small number of pianists, more precise evaluation of validity and reliability of the questionnaire items and scales should be performed. Finally, the present questionnaire could not control pieces related to occurrence of the choking, which however can be related to some factors of the choking, such as failure of memory recall that should matter when playing pieces relying heavily on working memory.

## Supporting information

S1 File (DOCX)

S2 File (DOCX)
