# Supplementary material for: Factors of choking under pressure in musicians
Source: PLoS One. 2021 Jan 6;16(1):e0244082. doi: 10.1371/journal.pone.0244082 (PMC7787383; doi:10.1371/journal.pone.0244082)
Supplement: S1 File — (DOCX) [file pone.0244082.s001.docx]

（１）Personal traits

1. I have a positive personality.
2. I have a passive personality.
3. I tend to feel guilty.
4. I make new friends easily.
5. I worry about what people say about me.
6. When I am doing something, I keep thinking about how others are judging me.
7. I am easily hurt.
8. I am often nervous or oversensitive.
9. I tend to worry about little things.
10. I get tired easily.
11. I tend to worry about things that I should not worry about.
12. I have a nervous temperament.
13. I get upset easily.
14. I tend to worry and get stressed about any number of things.
15. I tend to overcomplicate things.
16. A lot of things frequently bother me.
17. I don’t feel confident.
18. I always feel anxious about things and can’t relax.
19. I worry about things too much.
20. I get tense and irritated easily.
21. I am lively and energetic.
22. I am not good at talking in public.
23. I am quite gregarious.
24. I tend to be self-conscious when I am in public.
25. I have a submissive personality.
26. I am not afraid to express my opinions.
27. I am quite active and energetic.
28. I am a quiet person.
29. It takes a lot of effort for me to talk to new people.

（２）Experiencing choking under pressure (stage fright)

1. The way I moved and played kept getting worse and resulted in a poor performance.
2. I got discouraged with myself.
3. I could not move or perform correctly.
4. I failed to react quickly to an unexpected incident during my performance.
5. I felt embarrassed.
6. My stage fright increased because I failed to perform ideally.
7. My stage fright increased because I felt more tense after making a mistake.
8. I could not perform at an ideal level.
9. I was worried about failing.
10. I felt flustered as I was trying to calm down.
11. I was obsessed by a feeling of inferiority.
12. I tried to play confidently, which came off as being too strong.
13. I question myself about whether my performance was good or bad.
14. I was able to play without hesitation.
15. I was overwhelmed by the atmosphere.
16. I tried to deal with the situation but failed, which made my stage fright even worse.
17. I pushed down on the keyboard and pedals.
18. I could not relax.
19. I lost feeling in my arms and legs.
20. My breathing was erratic which made it hard to breathe.
21. I felt that my throat was choked.
22. My legs, arms, and fingers would not move the way they should do.
23. I felt afraid.
24. I felt like my hands were floating.
25. I felt like my center of gravity was rising.
26. I felt like my performance was sloppy and could not have been worse.
27. I felt that people’s eyes were boring into me.
28. I felt agitated.
29. The things around me all seemed wrong; for example, the keyboard seemed heavy; it felt like the eyes of audience were all focused on me; it felt like the audience was too close; the piano looked like it was on an incline; the lighting was dazzling; the seat was uncomfortable; and the keyboard seemed either too wide or too narrow.
30. My field of vision and the keyboard seemed different than usual.
31. I felt like the keyboard was either too heavy or too light.
32. I could not hear the sound well.
33. I could not feel the weight of the keyboard.
34. My arms and hands felt heavy.
35. I tried to make myself feel strength in my arms and legs, but it seemed like I had none.
36. I was worried about my posture and movements.
37. I tried hard to cope with the stage freight.
38. There were many occasions in which I hesitated.
39. I felt like my judgment was poor.
40. I played indecisively.
41. I was conscious of ways of behaving and playing that were less risky.
42. My performance was passive.
43. I was aware of the people I knew in the audience.
44. I was conscious of being in front of a big audience.
45. I felt aware of being judged by others.
46. I felt pressure.
47. I felt like I was in a situation where failure was not an option.
48. I felt nervous about hitting the wrong keys and losing my rhythm.
49. My mind was a blank and I could not recall memorized performance.（I completely forgot the music.）
50. I could not remember what I had memorized.
51. I was not able to play as I hoped.
52. My mind went blank.
53. I felt uneasy.
54. I felt rushed.
55. I couldn’t calm down.
56. I felt hot all over.
57. My arms and fingers (my body) felt stiff.
58. I was shaking.
59. I was worried about where I was looking during the performance.
60. My mouth and throat were dry.
61. I unintentionally sped up the tempo.
62. I was confused and it felt like the music just went out of control. (My fingers moved on their own.)
63. I was concerned about the parts that I was not good at playing.
64. I started thinking too much about the parts coming up later, which negatively impacted the entire performance.
