# Supplementary material for: Factors of choking under pressure in musicians
Source: PLoS One. 2021 Jan 6;16(1):e0244082. doi: 10.1371/journal.pone.0244082 (PMC7787383; doi:10.1371/journal.pone.0244082)
Supplement: S2 File — (DOCX) [file pone.0244082.s002.docx]

（１）性格特性

1. 前向きな性格である
2. 消極的である
3. 罪悪感を感じやすい
4. 新しい友人がすぐにできる
5. 自分についてのうわさが気になる
6. 他人からの評価を考えながら行動する
7. 傷つきやすい
8. よく緊張したり神経過敏になったりする
9. どうでもいいことを気に病む傾向がある
10. 疲れやすい
11. 自分で悩む必要のないことまで心配してしまう
12. 神経質である
13. 気持ちが動揺しやすい
14. あれこれ悩んだり，思いわずらったりしやすい
15. 物事を難しく考えがちである
16. 何かと気がかりなことが多い
17. いまひとつ自信がない
18. いつも気がかりなことがあって，落ち着かない
19. くよくよ考え込みやすい
20. 緊張してイライラしやすい
21. にぎやかな性格である
22. 人前で話すのは苦手だ
23. 積極的に人と付き合う方だ
24. 人前を気にする方だ
25. おとなしい性格である
26. 積極的に自分の意見を主張する
27. 活発に行動する方だ
28. 無口な方だ
29. 初対面の人と話をするのは骨が折れる

（２）演奏中のあがり経験

1. 良い演奏に結びつかない動き・弾き方が増えた
2. 自分に対して落胆した
3. 正確な動き・パフォーマンスができなかった
4. 演奏中に起きた想定外の事へのリアクション/対処が遅れた
5. 恥ずかしさを感じた
6. 思うような動作ができずに，‘あがり’がさらに促進していった
7. ミスをした後に不安が増えて，‘あがり’がさらに促進していった
8. 自分が理想とする演奏に結びつかなかった
9. 自分の失敗が気になった
10. 落ち着こうとして，かえって焦った
11. 劣等感にとらわれた
12. 思い切って弾こうとして，かえって力んだ
13. 自分のしている演奏が良いのか悪いのかわからなくなった
14. ためらうことなく演奏することができた
15. 雰囲気にのまれた
16. 対処しようとしたがうまくいかず‘あがり’が促進した
17. 鍵盤やペダルを押さえつけた
18. 脱力できなかった
19. 手足の感覚がわからなかった
20. 呼吸が乱れ，息苦しかった
21. 喉が詰まったような感じがした
22. 手足や指が思うように動かなかった
23. 恐怖心があった
24. 手が浮く感じがあった
25. 重心が上がっている感じがあった
26. 演奏の内容に関して，もうどうでも良いと投げやりな気になった
27. 他人の目が自分だけに向いている感じがした
28. イライラした
29. 周囲の物が，自分に対して不利に見えた （例:鍵盤が重そうに見えた，客席からの視線を強く感じた，客席が近い，ピアノが斜めに感じた，照明がまぶしいような気がした，椅子が合っていない，鍵盤の幅が狭く/広く感じる，など)
30. 視界や鍵盤が普段と違って見えた
31. 鍵盤が重く，あるいは軽く感じられた
32. 音が十分に聴こえなかった
33. 鍵盤の重さが感じられなかった
34. 手や腕が重たかった
35. 手足に力を入れようとしても力が入らなかった
36. 自分の姿勢や動作が気になった
37. ‘あがり’に対処しようと，努力した
38. ちゅうちょする（ためらう）場面が多かった
39. 判断力が低下した
40. 思い切りの無い演奏をしていた
41. 失敗のリスクが少ない動作・弾き方を意識した
42. 消極的な演奏をした
43. 知人の前であることを意識した
44. 大勢の人前であることを意識した
45. 他人に評価されていることを意識した
46. プレッシャーを感じた
47. 失敗の許されない場面であると感じた
48. ミスタッチやリズムがずれることへの不安を感じた
49. 暗譜がとんだ（曲を忘れた）
50. 覚えていたところが思い出せなかった
51. 自分の思ったとおりに演奏できなかった
52. 頭の中が真っ白になった
53. 不安を感じた
54. あせりを感じた
55. 落ち着かなかった
56. 身体が熱い感じがした
57. 指や腕（身体）がかたくなった
58. 身体が震えた
59. 演奏中に，自分がどこを見ているのかが気になった
60. 口や喉が渇いた
61. テンポが意図せず速くなった
62. 思考が混乱し，曲が先に先にと制御できず進んでしまった（指が勝手に動いた）
63. 苦手箇所が気になった
64. 今演奏している箇所よりもかなり先の箇所を意識し過ぎてしまい，演奏に好ましくない影響が出た
